# A Retrospective, Multicenter, Long-Term Follow-Up Analysis of the Prognostic Characteristics of Recurring Non-Metastatic Renal Cell Carcinoma After Partial or Radical Nephrectomy

**DOI:** 10.3389/fonc.2021.653002

**Published:** 2021-06-28

**Authors:** Sung Han Kim, Boram Park, Eu Chang Hwang, Sung-Hoo Hong, Chang Wook Jeong, Cheol Kwak, Seok Soo Byun, Jinsoo Chung

**Affiliations:** ^1^ Department of Urology, Urologic Cancer Center, Research Institute and Hospital of National Cancer Center, Goyang, South Korea; ^2^ Statistics and Data Center, Research Institute for Future Medicine, Samsung Medical Center, Seoul, South Korea; ^3^ Department of Urology, Chonnam National University Medical School, Gwangju, South Korea; ^4^ Department of Urology, Seoul St. Mary’s Hospital, Seoul, South Korea; ^5^ Department of Urology, Seoul National University College of Medicine and Hospital, Seoul, South Korea; ^6^ Department of Urology, Seoul National University Bundang Hospital, Seongnam, South Korea

**Keywords:** prognosis, nephrectomy, metastasis, renal cell carcinoma, recurrence

## Abstract

This study aimed to compare the cancer-specific survival (CSS) and overall survival (OS) of nephrectomized patients with non-metastatic renal cell carcinoma (nmRCC) and local recurrence without distant metastasis (LR group), those with metastasis without local recurrence (MET group), and those with both local recurrence and metastasis (BOTH group). This retrospective multicenter study included 464 curatively nephrectomized patients with nmRCC and disease recurrence between 2000 and 2012; the follow-up period was until 2017. After adjusting for significant clinicopathological factors using Cox proportional hazard models, CSS and OS were compared between the MET (n = 50, 10.7%), BOTH (n = 95, 20.5%), and LR (n = 319, 68.8%) groups. The CSS and OS rates were 34.7 and 6.5% after a median follow-up of 43.9 months, respectively. After adjusting for significant prognostic factors of OS and CSS, the MET group had hazard ratios (HRs) of 0.51 and 0.57 for OS and CSS (p = 0.039 and 0.103), respectively, whereas the BOTH group had HRs of 0.51 and 0.60 for OS and CSS (p < 0.05), respectively; LR was taken as a reference. The 2-year OS and CSS rates from the date of nephrectomy and disease recurrence were 86.9% and 88.9% and 63.5% and 67.8%, respectively, for the LR group; 89.5% and 89.5% and 48.06% and 52.43%, respectively, for the MET group; and 96.8% and 96.8% and 86.6% and 82.6%, respectively, for the BOTH group. Only the LR and BOTH groups had significant differences in the 2-year OS and CSS rates (p < 0.05). In conclusion, our study showed that the LR group had worse survival prognoses than any other group in nephrectomized patients with nmRCC.

## Introduction

Globally, the number of incidentally diagnosed localized non-metastatic renal cell carcinomas (nmRCCs) has increased due to improvements in diagnostic modalities (1). Given that the radical removal of primary RCC by partial or complete nephrectomy is the standard treatment for nmRCC, approximately 7–30% of surgically treated RCCs recur within 5 years (2), and another 20–40% of RCCs progress to metastasis after curative surgery, resulting in a poor 5-year overall survival (OS) of <20% (3–5). Both clinicians and researchers have attempted multiple times to overcome the diverse and unpredictable survival outcomes of local recurrence (LR) and distant metastasis in patients with nmRCC after nephrectomy, and various definitions of disease recurrence in multiple cohorts have shown different prognostic outcomes (4–7). Several predictive factors of OS and CSS, such as the interval between nephrectomy and LR or metastasis development, the characteristics of recurrent or metastatic tumors, and the different pathological and genetic backgrounds of primary tumors, have been suggested (5–7). However, some guidelines recommend a 5-year follow-up period, which is not adequate to manage RCC as it either presents with delayed LR or only as metastasis without LR (MET) in approximately 5–10% of patients, even after a 5-year disease-free period, due to its heterogenetic, intratumoral, and distinct histological characteristics (4–6). Therefore, researchers have put extensive efforts for several decades into finding significant predictive markers for LR and MET in RCC, after either radical or partial nephrectomy. Such markers can predict patients with a high risk of LR and MET after nephrectomy, even with clear resected margins.

This study aimed to assess the predisposing characteristics and survival prognoses of patients with LR and no metastasis (LR group), those with metastasis and no LR (MET group), and those with LR and metastasis (BOTH group). The data of 464 patients who underwent RCC nephrectomy with postoperative disease recurrence were collected retrospectively from six Korean institutions. The patients in this study either underwent nmRCC radical or partial nephrectomy with a follow-up period until the end of 2017. Survival prognosis analysis focused on the OS and cancer-specific survival (CSS) for all groups.

## Materials and Methods

### Ethical Statement

This retrospective study was approved by the institutional review board of the National Cancer Center (approval number: NCC 2018–0045 and B1202/145-102), which waived the requirement for informed consent due to the retrospective nature of this study (8–10). All study procedures were performed in accordance with the tenets of the ethical guidelines and regulations of the Ethical Principles for Medical Research Involving Human Subjects of the World Medical Association Declaration of Helsinki.

### Patient Criteria and RCC Database

Data of the 4,246 enrolled patients with RCC were obtained from two multicenter RCC databases—the nmRCC (8) and mRCC (9) databases—that were obtained from the Multicenter Korean National Kidney Cancer (MKNKC) database. A total of 464 (10.9%) patients with RCC were selected for this study. All participants underwent curative partial or radical nephrectomy with or without lymphadenectomy between 2000 and 2012 and attended at least a follow-up period of 1 month until either local recurrence or distant metastasis was detected. Exclusion criteria were age <19 years (n = 12); histologically confirmed benign tumor (n = 73); postoperative disease recurrence within 1–3 months to exclude obscured synchronous metastasis at nephrectomy (n = 5); positive resection margins after partial/radical nephrectomy (n = 35); and a history of cytoreductive nephrectomy, incomplete medical records of survival outcomes, a history of previous cancers, and same patient visiting different hospitals (n = 73).

The parameters analyzed in this study were baseline anthropometric characteristics, including age, sex, and underlying diseases; preoperative baseline laboratory findings, including serum albumin, hemoglobin, and creatinine; intraoperative nephrectomy information; pathology results, including pTNM stage, histology, Fuhrman nuclear grade, sarcomatoid differentiation, lymphovascular invasion, necrosis, and capsule invasion; and survival outcomes, including all-cause and cancer-specific deaths. The surgical procedures of partial and radical nephrectomies were documented in a previous study (8–10); however, no specific standardized protocol was followed for surgical procedures during the collection of data for the RCC database. For the initial postoperative imaging follow-up, imaging intervals, that is, either 1- or 3-month intervals, were not standardized and were based on the preference of the urologist for the postoperative surveillance protocol established at the time.

### Patient Classification

Patients were categorized into the LR (n = 319, 68.8%), MET (n = 50, 10.7%), and BOTH (n = 95, 20.5%) groups. The LR group comprised nephrectomized patients with clear resection margins who were diagnosed with local recurrence at the renal fossa without distant metastasis throughout the postoperative 1–3-month imaging follow-up, whereas the MET group comprised only those with post-nephrectomy distant metastasis without LR around the renal fossa throughout the 1–3-month postoperative follow-up period. The BOTH group comprised nephrectomized patients diagnosed with only postoperative LRs in the operated renal fossa who later experienced disease progression in the distal metastatic organs. The BOTH group included four patients who were simultaneously diagnosed with local recurrence and distant metastasis.

### Statistical Analysis

Baseline characteristics are presented as frequencies (percentages) for categorical variables and as medians [interquartile range (IQR) or mean ± standard deviations (SD)] for continuous variables. Differences in distributions were compared among the three groups using a one-way analysis of variance or the Kruskal-Wallis test for continuous variables and Pearson’s χ^2^ test or Fisher’s exact test for categorical variables. A *post-hoc* analysis was performed to explore the clinicopathological factors that differed among the LR, MET, and BOTH groups. Given that we performed two analyses among the three groups, we set the significance cut-off at 0.05/2 for the *post-hoc* tests, taking multiple comparisons into consideration.

The survival indices OS and CSS were used to assess all-cause and RCC-related deaths, respectively. OS and CSS were compared among groups using the Cox proportional hazard models after adjusting for important covariates. A backward variable selection method with an elimination criterion of P > 0.05 was performed to complete the multivariable model. Survival curves were plotted using the survival probabilities of a multivariable model, and the survival rates of the three groups from 1 to 15 years were calculated using the Kaplan-Meier method. A two-sided p-value < 0.05 was considered statistically significant. Statistical analysis was performed using SAS 9.4 (SAS Institute Inc., Cary, NC, USA).

### Data Availability

The datasets generated and/or analyzed during the current study are available to be provided from the corresponding author upon reasonable request.

## Results

Throughout the median follow-up of 43.9 (range, 19.0–76.1) months, the local recurrence, metastasis, and mortality rates following nephrectomy were 68.7, 31.3, and 41.2%, respectively, including 161 (34.7%) and 30 (6.5%) RCC-related deaths and non-RCC-related deaths, respectively. Preoperative serum platelet and albumin levels, operative methods, and pathologic N stage were significantly different clinicopathological factors among the three groups (p < 0.05, **Table 1**). Moreover, baseline platelet levels and the nephrectomy method were significantly different between the LR and MET groups (**Supplementary Table 1A**), and the baseline albumin levels, pN1 stages, and intratumor necrosis characteristics were significantly different between the LR and BOTH groups (p < 0.05, **Supplementary Table 1B**).

**Table 1:** Baseline characteristics (N = 464).

		Total	LR group	MET group	BOTH group	p-value
Number		464	319	50	95	
Follow-up duration	median (IQR)	93.2 (48.4–127.7)	93.2 (48.4–127.7)	36.8 (13.8–100.9)	93.2 (49.6–124.5)	
Age at operation	mean ± STD	56.5 ± 11.6	56.5 ± 11.6	59.4 ± 9.9	56.7 ± 11	0.238
Body mass index (kg/cm2)	mean ± STD	23.9 ± 3	23.9 ± 3	24.5 ± 3.5	24 ± 2.9	0.481
Diabetes	yes	47 (14.7)	47 (14.7)	8 (16)	13 (13.7)	0.929
Hypertension	yes	109 (34.2)	109 (34.2)	24 (48.0)	42 (44.2)	0.059
Hemoglobin	median (IQR)	13.3 (11.4–14.6)	13.3 (11.4–14.6)	13.3 (11.6–14.6)	13.8 (12.2–14.9)	0.348
Platelet	median (IQR)	255.5 (212–318.5)	255.5 (212–318.5)	212 (181–261)	251 (212–299)	0.005
Creatinine	median (IQR)	1 (0.9–1.2)	1 (0.9–1.2)	1 (0.83–1.2)	1 (0.9–1.16)	0.916
Albumin	median (IQR)	4.1 (3.7–4.4)	4.1 (3.7–4.4)	4.2 (3.8–4.4)	4.2 (3.9–4.5)	0.034
Nephrectomy	Open surgery	328 (70.7)	233 (73.0)	23 (46.0)	72 (75.8)	<.001
	Laparoscopic	127 (29.3)	83 (26.0)	26 (52.0)	18 (19.0)	
Operative Extent	partial	58(12.5)	41 (12.9)	7 (14.0)	10 (10.5)	0.994
	radical	196 (42.2)	140 (43.9)	23 (46.0)	33 (34.7)	
pathologic T stage	T1	138 (43.3)	138 (43.3)	19 (38.0)	31 (32.6)	0.568
	T2	57 (17.9)	57 (17.9)	10 (20.0)	23 (24.2)	
	T3	111 (34.8)	111 (34.8)	19 (38.0)	36 (37.9)	
	T4+Tx	12 (3.8)	12 (3.8)	1 (2.0)	2 (2.1)	
pathologic N stage	N0+Nx	291 (91.2)	291 (91.2)	45 (90.0)	92 (96.8)	0.015
	N1	27 (8.5)	27 (8.5)	4 (8.0)	0 (0.0)	
Nuclear grade	grade 1-2	82 (25.7)	82 (25.7)	13 (26.0)	20 (21.1)	0.206
	grade 3-4	147 (46.1)	147 (46.1)	31 (62.0)	59 (62.1)	
Sarcomatoid differentiation	yes	13 (4.1)	13 (4.1)	2 (4.0)	8 (8.4)	0.269
Necrosis	yes	43 (13.5)	43 (13.5)	8 (16.0)	22 (23.2)	0.075
Lymphovascular invasion	yes	39 (12.2)	39 (12.2)	10 (20.0)	7 (7.4)	0.084
Capsular invasion	yes	72 (22.6)	72 (22.6)	17 (34.0)	22 (23.2)	0.208
Cause of death (n = 191)	RCC related	117 (80.7)	117 (80.7)	11 (91.7)	33 (97.1)	
	non-RCC-related	28 (19.3)	28 (19.3)	1 (8.3)	1 (2.9)	

**Supplementary Table 2** describes the analysis of the predictive clinicopathological factors of OS and CSS in each group. Multivariate analysis results (**Supplementary Table 2**) showed that the body mass index, hypertension, hemoglobin and albumin levels, pT3-4 and pN1 stageS, and Fuhrman nuclear grades 3–4 were significant risk factors of OS (p < 0.05), whereas body mass index, diabetes, hypertension, hemoglobin and albumin levels, pT3-4 and pN1 stageS, and Fuhrman nuclear grades 3–4 were the risk factors of CSS (p < 0.05). After adjusting for the significant risk factors of OS and CSS, the MET group had a significant hazard ratio (HR) of 0.51 (95% confidence interval [CI]: 0.27−0.97) for OS (p = 0.039) and an insignificant HR of 0.57 (CI: 0.29−1.12) for CSS (p = 0.103). The BOTH group had HRs of 0.51 (95% CI: 0.27–0.77) and 0.60 (95% CI: 0.39–0.92) for OS and CSS (p < 0.05), respectively, with the LR group (HR, 1.0) as a reference (**Table 2**).

**Table 2:** Univariable and multivariable Cox proportional hazard models for overall survival (OS) and cancer-specific survival (CSS) among three groups.

Group	Metastasis	Recurrence	Total	Event (%)	Univariable model		Multivariable model	
					HR (95% CI)	p-value	HR (95% CI)	p-value
**(1) Overall Survival (OS)**								
LR group	no	yes	319	145 (45.5)	1 (ref)	(0.0187)	1 (ref)	(0.0017)
MET group	yes	no	50	12 (24.0)	0.63 (0.35–1.15)	0.1326	0.51 (0.27–0.97)	0.0389
Both group	yes	yes	95	34 (35.8)	0.61 (0.42–0.89)	0.0104	0.51 (0.34–0.77)	0.0015
**(2) Cancer-Specific Survival (CSS)**								
LR group	no	yes	319	117 (36.7)	1 (ref)	(0.2405)	1 (ref)	(0.0302)
MET group	yes	no	50	11 (22.0)	0.73 (0.39–1.36)	0.3147	0.57 (0.29–1.12)	0.1026
Both group	yes	yes	95	33 (34.7)	0.74 (0.50–1.10)	0.1365	0.60 (0.39–0.92)	0.0209

Adjusted for body mass index, hypertension, hemoglobin, albumin, pT stage, pN stage, and nuclear grade in OS multivariable model.

CI, confidence interval.

**Table 3** describes the multivariate analyses of significant clinicopathological data within each group. Only capsular invasion was found to be a significant risk factor for both OS (HR: 8.97, CI: 1.83−44.09) and CSS (HR: 7.36, CI: 1.45–37.35) in the MET group (p < 0.05) in the subgroup analyses for selecting high-risk factors of OS and CSS. In the LR group, body mass index and preoperative hemoglobin and albumin levels were favorable risk factors of OS and CSS, whereas hypertension and pathologic T3-4 and N1 stages were unfavorable risk factors of both OS and CSS; a Fuhrman nuclear grade 3-4 was a risk factor of CSS only (p < 0.05). In the BOTH group, diabetes, lymphovascular invasion, and a Fuhrman nuclear grade 3-4 were significant factors of both OS and CSS (p < 0.05).

**Table 3:** Multivariable Cox proportional hazard model in each subgroup for overall survival (OS) and cancer-specific survival (CSS).

			Overall survival (OS)		Cancer-specific survival (CSS)	
			HR (95% CI)	p-value	HR (95% CI)	p-value
MET group			n = 50, event = 12 (24.0%)		n = 50, event = 11 (22.0%)	
	Capsular invasion	yes	8.97 (1.83–44.09)	0.007	7.36 (1.45–37.35)	0.016
				
LR group			n = 304, event = 132 (43.4%)		n = 319, event = 117 (36.7%)	
	Body mass index (kg/cm2)		0.89 (0.84–0.95)	<.001	0.87 (0.81–0.94)	<.001
	Hypertension	yes	2.07 (1.41–3.02)	<.001	2.78 (1.80–4.30)	<.001
	Hemoglobin	female (≤12), male (≤13)	1 (ref)		1 (ref)	
		female (>12), male (>13)	0.45 (0.30–0.68)	<.001	0.47 (0.30–0.74)	0.001
	Albumin	≤3.0	1 (ref)		1 (ref)	
		>3.0	0.21 (0.09–0.52)	<.001	0.20 (0.08–0.50)	0.001
	pathologic T stage	T1	1 (ref)	(<.001)	1 (ref)	(<.001)
		T2	1.30 (0.78–2.19)	0.318	1.69 (0.92–3.08)	0.089
		T3	1.98 (1.30–3.03)	0.002	2.31 (1.42–3.76)	0.001
		T4+Tx	9.41 (4.02–22.02)	<.001	11.4 (4.66–27.86)	<.001
	pathologic N stage	N0+Nx	1 (ref)		1 (ref)	
		N1	2.21 (1.18–4.13)	0.014	2.63 (1.35–5.13)	0.005
	Nuclear grade	grade 1-2			1 (ref)	
		grade 3-4			1.92 (1.09–3.38)	0.024
				
BOTH group			n = 95, event = 34 (35.8%)		n = 95, event = 33 (34.7%)	
	Diabetes	yes	2.69 (1.17–6.18)	0.02	3.03 (1.30–7.07)	0.011
	Lymphovascular invasion	yes	4.19 (1.41–12.46)	0.01	4.01 (1.32–12.22)	0.015
	Nuclear grade	grade 1-2			1 (ref)	
		grade 3-4			3.35 (1.11–10.08)	0.032

CI, confidence interval.

When the 2-year and 3-year survival rates from the nephrectomy date were analyzed in the three groups, the OS and CSS rates were 89.5% and 79.4% and 89.5% and 83.2%, respectively, for the MET group; 86.9% and 80.3% and 88.9% and 82.9%, respectively, for the LR group; and 96.8% and 93.2% and 96.8% and 93.2%, respectively, for the BOTH group (**Table 4A**). Only the LR and BOTH groups had significant differences in the 2- and 3-year OS and CSS rates (p < 0.05). Considering the starting date of disease recurrence, the 2-year and 3-year survival rates of OS and CSS rates were 48.1% and 48.1% and 52.4% and 52.4%, respectively, for the MET group; 63.5% and 57.3% and 67.8% and 61.8%, respectively, for the LR group; and 82.6% and 71.6% and 82.6% and 71.6%, respectively, for the BOTH group (**Table 4B**). Only the 2-year OS and CSS rats were significantly different between the LR and BOTH groups (p < 0.05).

**Table 4:** Survival rate of 2 and 3 years according to three groups **(A)** from the date of nephrectomy and **(B)** from the date of local recurrence or metastasis.

Group	N	Overall survival						Cancer-specific survival					
		2 years	95% CI		3 years	95% CI		2 years	95% CI		3 years	95% CI	
		survival rate	Lower	Upper	survival rate	Lower	Upper	survival rate	Lower	Upper	survival rate	Lower	Upper
**(A) Survival rates according to three groups from the date of nephrectomy**													
LR	319	86.90%	83.15%	90.80%	80.30%	75.85%	85.10%	88.90%	85.40%	92.60%	82.90%	78.60%	87.40%
MET	50	89.50%	80.20%	99.90%	79.40%	66.60%	94.60%	89.50%	80.17%	99.90%	83.20%	71.61%	96.60%
BOTH	95	96.80%	93.22%	100.0%	93.20%	88.17%	98.60%	96.80%	93.22%	100.0%	93.20%	88.17%	98.60%
**(B) Survival rates according to three groups from the date of local recurrence or metastasis**													
LR	319	63.50%	57.70%	69.80%	57.30%	51.20%	64.00%	67.80%	62.10%	74.10%	61.80%	55.70%	68.60%
MET	50	48.06%	29.20%	79.00%	48.06%	29.20%	79.00%	52.43%	32.90%	83.60%	52.43%	32.90%	83.60%
BOTH	95	82.60%	74.40%	91.70%	71.60%	61.60%	83.10%	82.60%	74.40%	91.70%	71.60%	61.60%	83.10%

CI, confidence interval.

**Figure 1:**
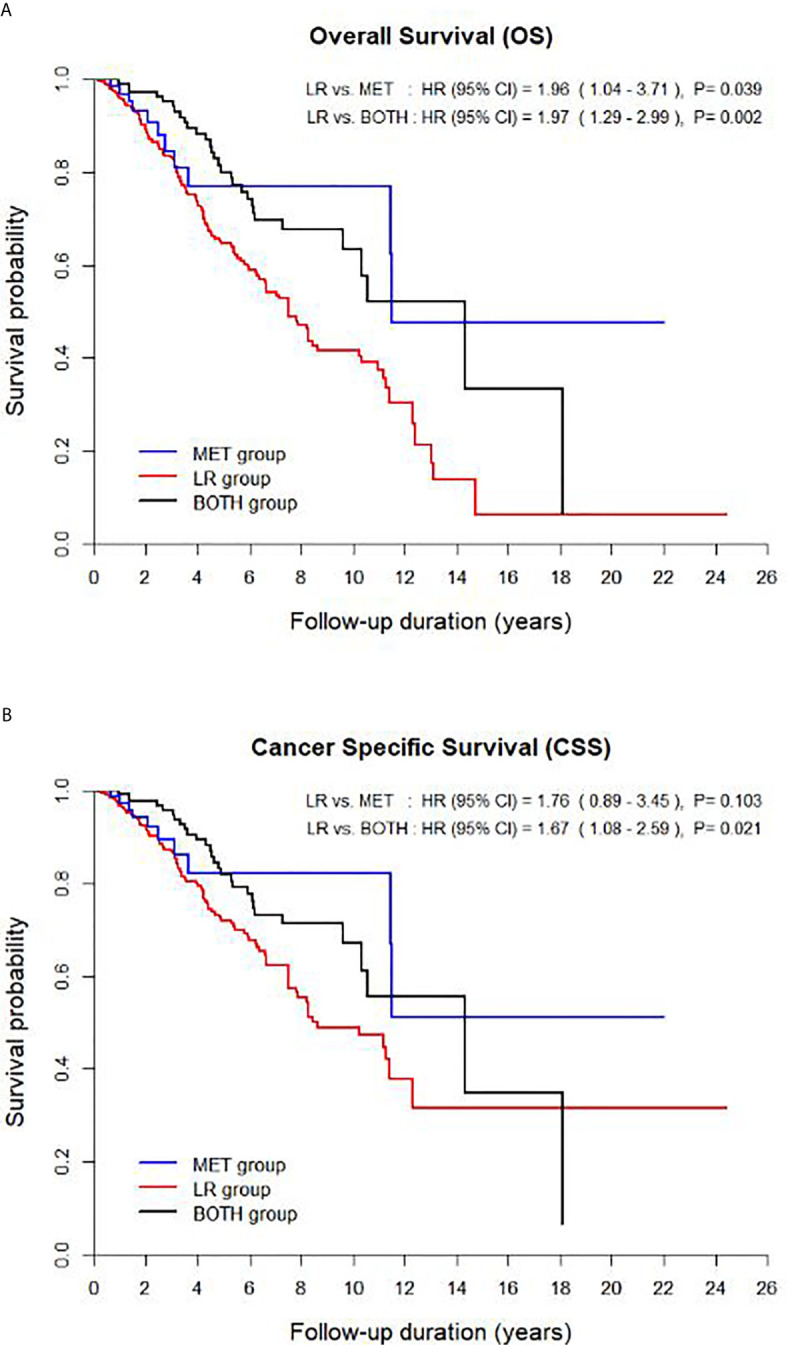
Survival curves of the multivariable model for **(A)** overall survival and **(B)** cancer-specific survival among the three groups. CI, confidence interval.

The OS and CSS curves adjusted for significant covariates showed significant differences among the three groups (p < 0.005, **Figure 1**). There was a significant difference in OS between the LR group and each of the other two groups (*versus* MET, HR 1.96, and *versus* BOTH, HR 1.97) (p < 0.05, **Figure 1A**). However, only the BOTH group significantly differed in CSS from the LR group (LR *versus* BOTH, HR 1.67) (p = 0.021, **Figure 1B**).

**Supplementary Table 3** shows the extended long term-survival rates spanning up to 15 years postoperatively. A significant difference in both OS and CSS was observed between the LR and BOTH groups at 2–3 years postoperatively, whereas a significant difference in only OS was observed between the aforementioned groups at 4 and 9 years postoperatively (p < 0.05). There was a significant difference in both OSS and CSS between the MET and LR groups at 9, 10, and 11 years postoperatively, whereas there was a significant difference in only OS between the aforementioned groups at 8 years postoperatively. These differences remained in all groups until 15 years postoperatively (all, p < 0.05).

## Discussion

Disease recurrence after curative nephrectomy in patients with nmRCC is challenging due to its rarity and unpredictability owing to the heterogenetic and pleomorphic pathophysiology of RCC, making large prospective studies, including randomized controlled trials, rare and inducing conflicting issues related to therapeutic and follow-up guidelines. These limitations allow retrospective multicenter studies comprising large study samples, such as in this study, to define significant independent disease recurrence predictive factors and characterize the prognostic survival of patients with nmRCC after nephrectomy (1–4, 11–14). This study selected a sufficient number of post-nephrectomized patients with recurrence and stratified them into LR, MET, and BOTH groups according to their recurrence patterns. Significant independent predictive and prognostic risk factors of OS and CSS were found, and some important characteristic findings regarding disease recurrence were obtained to improve postoperative surveillance and therapeutic strategic information.

**Figure 2:**
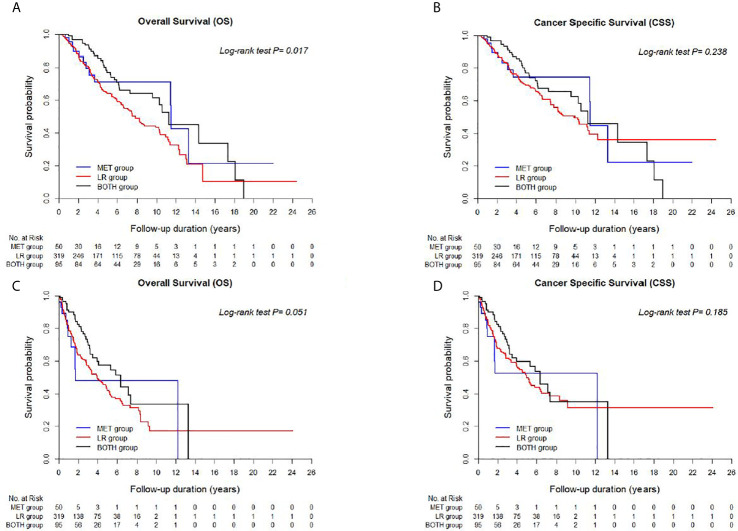
**** Kaplan-Meier curves between LR, BOTH, and MET groups for **(A)** Overall survival (OS) and **(B)** Cancer-specific survival (CSS) from the date of nephrectomy, and **(C)** OS and **(D)** CSS from the date of disease recurrence or metastasis.

The comparison of prognostic survival among the three groups demonstrated that both metastatic groups (HR < 1.0 for OS and CSS, **Table 2**) had significantly better OS and CSS than the LR group; however, there was an insignificant difference in CSS between the LR and MET groups (**Figures 1**, **2**). Nevertheless, there were no significant differences in OS and CSS between both the MET and BOTH groups (p > 0.05, **Table 2**). Moreover, the 2- and 3-year OS and CSS rates supported the aforementioned unfavorable prognostic outcomes of the LR group, as well as the fact that the LR and BOTH groups had significantly different 2-year OS and CSS, regardless of the time from nephrectomy to disease recurrence (p < 0.05, **Table 4**). These results were unexpected and different from the general concepts of the survival of patients with RCC, which state that patients with mRCC had poorer survival outcomes than those with locally recurrent RCC (**Table 2**). This may be due to the distinguishing characteristics of this cohort compared to those in other studies (4, 5, 11–18). This study excluded patients who unsuccessfully underwent nephrectomy and have residual tumor cells at the renal fossa and those with obscured synchronous mRCCs who were at high risk of disease recurrence with suspicions of high tumor extents and aggressive tumor burdens. Moreover, this study includes a higher proportion of early stage patients with small tumor sizes (T1-staged RCC, 84.7%) and young patients (55.5 ± 12.4 years) compared to other studies. The characteristics of this cohort allowed us to focus on the primary tumor and disease progression, that is, either isolated local recurrence or distant metastasis, resulting in a lower number of patients in the MET (n = 50) and BOTH (n = 95) groups than in the LR group (n = 319) (3–5). The higher rate of early stage patients and small-sized tumors and the lower rate of high stage patients and large-sized tumors in this study were due to the fact that the Korean National Cancer Screening Program performs testing twice at the age of 40 and 55 years, significantly affecting the survival outcomes of each group compared to previous studies (**Table 1**) (3–6, 11–18).

Another explanation for the differential survival outcomes among groups was the differential characteristics of the LR group, which included more advanced infiltrating diseases, poorer general conditions, and higher tumor burdens requiring more open surgery than the remaining two groups (**Tables 1**, **3** and **Supplementary Table 1** and **Figure 1**). The higher baseline platelet levels and open surgery rate in the LR group than in the MET group and the more frequent nodal positivity and less necrotic primary lesions with lower albumin levels in the LR group than in the BOTH group supported the unfavorable prognoses of this group (p < 0.05, **Supplementary Table 1**) (14, 15, 19). Moreover, the therapeutic modalities of the LR and other groups were also important prognostic factors. This study did not discuss the therapeutic modalities of disease recurrence, but another Korean population-based study studying the therapeutic trends of disease recurrence (4.4%) after radical nephrectomy in 25,792 patients with nmRCCs between 2007 and 2013 showed significantly different OS rates between surgical methods (30.4 ± 18.7 months) and significantly different recurrence rates between targeted therapies (31 ± 22 months), other systemic therapies (25.4 ± 21.1 months), and radiation (24.1 ± 22.3 months) therapies. Therefore, it is possible to identify patients at higher risk in the LR group who may need closer follow-ups and earlier consideration for various adjuvant therapeutic strategies including intervention measures according to recurrent sites compared to low-risk patients.

As for the predictive factors of OS and CSS in each group, this study found that baseline hypertension, pathologic T3-4, N1 staging, and Fuhrman nuclear grade 3-4 are significant unfavorable risk factors and that high body mass index and hemoglobin and albumin levels are significant favorable risk factors in the LR group (p < 0.05, **Table 3**) (16–18, 20). Capsular invasion in the MET group and diabetes, lymphovascular invasion, and high nuclear grade in the BOTH group were also important prognostic factors of poor OS and CSS (p < 0.05, **Table 4**) (4, 21, 22). These independent prognostic factors were clinically important to stratify high-risk patients with poor prognoses at recurrence diagnosis during follow-up. Moreover, the post-nephrectomized 2-year and 3-year follow-ups from the time of disease recurrence were important time points to compare the survival rates between groups (p < 0.05, **Table 4**). These data suggest that high-risk patients with diseased recurrence should be monitored more closely within 2 years, meaning that survival prognoses were determined within 2 years and that the more active and earlier administration of adjuvant therapies should be considered to improve survival outcomes. Therapeutic recommendations for LR lesions should be established according to the location, extent, and size of tumor in each recurrence as non-established guidelines, definitions, and recommendations for LR allow various therapies based on the discretion of clinicians, resulting in inconsistent prognostic results (1–5).

As for surgical or interventional LR measures, several studies have reported that diseases progressed in 40–60% of patients following therapeutic measures, even in a nephrectomized patient with nmRCC and an isolated LR, implying that survival improved following measures (11–14). Bruno et al. (11) reported a 2.9% overall LR (LR: 1.5% and BOTH: 1.4%) in 1165 pT1-4N0M0-staged nephrectomy patients during a median time of 16.9 months (range, 0.5−103.6). Surgical intervention ensured a good OS and 3-year survival rates of 37.5 and 31%. Itano et al. (13) reported a disease recurrence rate of 2.9% (LR: 1.8%, and BOTH: 1.1%) in 1,737 pT1-3N0M0-staged radical nephrectomy patients. The disease control rate of surgical intervention was estimated at 60% of the OS rate. Margulis et al. (14) reported an LR rate of 1.8% during a median follow-up of 42 months in 2,945 pT1-3N0M0-staged nephrectomy patients. Surgical intervention ensured disease recurrence and overall mortality rates of 2.0% (27 distant metastases and 8 isolated LRs) and 1.8%, respectively. Therefore, further suggestions for effective surgical guidelines for LR and metastatic group indications should be discussed considering the association between metastases and other interventions and systemic therapy. Moreover, the surveillance of therapeutic strategies with close monitoring should also be considered within 2 years of recurrence until 4 years based on the type of metastasis (**Table 2** and **Supplementary Table 3**).

There is no consensus on preventive adjuvant systemic therapies and on when to apply adjuvant systemic therapies for disease recurrence after nephrectomy in patients with nmRCC because of contradicting results between previous clinical trials in this era of targeted therapy. The S-TRAC (sunitinib, positive), PROTECT (pazopanib), ARISER (girentuximab), and ATLAS (axitinib) trials showed contradicting results regarding the efficacy of adjuvant targeted therapies in a specified subset of nephrectomized patients with nmRCC (23, 24). In the upfront immune therapy era, several new ongoing trials showed that adjuvant immune-checkpoint inhibitors were efficient in nephrectomized patients with nmRCC (Keynote 564 trial, NCT03142334) (25), contradicting previous trials in the targeted therapy era. Immune checkpoint inhibitors potentiate systemic immune responses to the remnant cancer cells in secondary tumor sites after the complete removal of the primary kidney tumor (26). Some suggestions for future trials include investigating the effects of combining an immune-checkpoint inhibitor with targeted therapy and localized intervention for controlling disease recurrence and microtumor environments (26, 27).

Lastly, regarding the choice of surgical treatment, that is, radical or partial nephrectomy, and surgical technique, that is, open or laparoscopic surgery, survival was not influenced by nephrectomy itself, especially in early stage patients with confined nmRCC. Open and radical nephrectomy reportedly often showed poorer prognostic outcomes than other techniques and also disease recurrence because open nephrectomy was more suitable for patients with advanced stage tumors, as well as nodal infiltration, high intratumor burdens, poor preoperative characteristics and immunity, and a high likelihood of increased circulating cancer cells *via* the lymphovascular system intraoperatively, resulting in a higher probability of disease recurrence/progression after nephrectomy compared to other techniques (28–30). Selecting appropriate patients with nmRCC for nephrectomy is important to successfully remove all tumor cells to reduce disease recurrence.

This study had several inherent limitations due to its retrospective multicenter design, missing values, and missing information on postoperative prognoses, such as non-standardized surgical procedures, treatment modalities, specific locations, and disease recurrence criteria, and tumor burdens. However, only a few studies with large cohorts were available to characterize disease recurrence and predict prognostic factors after nephrectomy in patients with nmRCC. The findings from this study, along with several significant factors in each group, may help identify high-risk patients with nmRCC and better manage LR and MET with adequate follow-ups and therapeutic plans after nephrectomy. Future trials on postoperative preventive measures, on the determination of risk factors in patients with LR that can progress to distant metastasis and those who had the best survival outcomes, and on adjuvant therapy should be conducted.

This retrospective, multicenter, long-term follow-up nmRCC study showed that the LR group had worse survival prognoses than the remaining recurrent metastatic groups. The independent risk factors of survival in each group may indicate other high risk disease recurrence factors that may require adjuvant systemic therapies and local therapy to improve the prognosis of patients with nmRCC after either radical or partial nephrectomy. Further prospective cohort studies should be conducted to validate our findings and provide suggestions for LR and metastatic groups.

## Data Availability Statement

The original contributions presented in the study are included in the article/**Supplementary Material**. Further inquiries can be directed to the corresponding author (cjs5225@ncc.re.kr).

## Ethics Statement

The studies involving human participants were reviewed and approved by National Cancer Center, Korea. Written informed consent for participation was not required for this study in accordance with the national legislation and the institutional requirements.

## Author Contributions

SK: Conceptualization, data curation, investigation, methodology, project administration, supervision, and writing (original draft preparation). EH, S-HH, CJ, CK, and SB: Conceptualization, data curation, investigation, methodology, supervision, and writing (original draft preparation). BP: Conceptualization, data curation, formal analysis, investigation, methodology, project administration, supervision, and writing (original draft preparation). JC: Conceptualization, data curation, investigation, methodology, project administration, supervision, funding acquisition, and writing (original draft preparation). All authors contributed to the article and approved the submitted version.

## Funding

This study was supported by the funding grant from the National Cancer Center (approval number: NCC 1710290-3).

## Conflict of Interest

The authors declare that the research was conducted in the absence of any commercial or financial relationships that could be construed as a potential conflict of interest.

## Supplementary Material

Supplementary Table 1 Comparison of baseline clinicopathological characteristics **(A)** between the LR and the MET and **(B)** between the LR and the BOTH groups

Supplementary Table 2 Univariable and multivariable Cox proportional hazard models between groups for overall survival (OS) and cancer-specific survival (CSS)

Supplementary Table 3 Survival rates for overall survival (OS) and cancer-specific survival (CSS) from 1 years to 15 years according to three groups
