# Supplementary material for: A Retrospective, Multicenter, Long-Term Follow-Up Analysis of the Prognostic Characteristics of Recurring Non-Metastatic Renal Cell Carcinoma After Partial or Radical Nephrectomy
Source: Front Oncol. 2021 Jun 28;11:653002. doi: 10.3389/fonc.2021.653002 (PMC8273547; doi:10.3389/fonc.2021.653002)
Supplement: Supplementary Table 1 — Comparison of baseline clinicopathological characteristics (A) between the LR and the MET and (B) between the LR and the BOTH groups [file DataSheet_1.pdf]

Supplementary table 1. Comparison of baseline clinicpoathological characteristics (A) between the LR and the MET and (B) between the LR and the BOTH groups

(A) Comparison between the LR and the MET groups

		MET group	LR group	P-value
Number		50	319	
Hypertension	yes	24 (48.0)	109 (34.2)	0.058
Platelet	median (IQR)	212 (181-261)	255.5 (212-318.5)	0.001
Nephrectomy	Open surgery	23 (46.0)	233 (73.0)	<.001
	Laparoscopic	26 (52.0)	83 (26.0)	
Capsular invasion	yes	17 (34.0)	72 (22.6)	0.079

(B) Comparison between the LR and the Both groups

		LR group	BOTH group	P-value
Number		319	95	
Albumin	median (IQR)	4.1 (3.7-4.4)	4.2 (3.9-4.5)	0.011
pN	N0+Nx	291 (91.2)	92 (96.8)	0.004
	N1	27 (8.5)	0 (0.0)	
Necrosis	yes	43 (13.5)	22 (23.2)	0.023

Supplementary Table 2. Univariable and multivariable Cox proportional hazard models between groups for overall survival (OS) and cancer-specific survival (CSS)

		Overall survival (OS) total = 464, events = 191				Cancer-specific survival (CSS) total = 464, events = 161			
		Univariable model		Multivariable model		Univariable model		Multivariable model	
		HR (95% CI)	p-value	HR (95% CI)	p-value	HR (95% CI)	p-value	HR (95% CI)	p-value
Group	LR group	1 (ref)	(0.019)	1 (ref)	(0.0017)	1 (ref)	(0.241)	1 (ref)	(0.030)
	MET group	0.63 (0.35-1.15)	0.133	0.51 (0.27-0.97)	0.0389	0.73 (0.39-1.36)	0.315	0.57 (0.29-1.12)	0.103
	Both group	0.61 (0.42-0.89)	0.010	0.51 (0.34-0.77)	0.0015	0.74 (0.50-1.10)	0.137	0.60 (0.39-0.92)	0.021
Age at operation		1.02 (1.01-1.03)	0.007			1.02 (1.00-1.03)	0.020		
Body mass index (kg/cm2)		0.89 (0.85-0.94)	<.001	0.88 (0.84-0.93)	<.0001	0.89 (0.84-0.94)	<.001	0.87 (0.81-0.92)	<.001
Diabetes	yes	1.43 (0.98-2.08)	0.063			1.58 (1.06-2.36)	0.024	1.67 (1.05-2.65)	0.030
Hypertension	yes	1.29 (0.96-1.73)	0.090	2.31 (1.65-3.25)	<.0001	1.42 (1.03-1.94)	0.032	2.47 (1.68-3.63)	<.001
Hb	female (≤12), male (≤13)	1 (ref)		1 (ref)		1 (ref)		1 (ref)	
	female (>12), male (>13)	0.46 (0.34-0.63)	<.001	0.59 (0.42-0.83)	0.0027	0.43 (0.30-0.60)	<.001	0.58 (0.4-0.84)	0.004
Platelet	≥150, ≤450	1 (ref)				1 (ref)			
	<150	0.40 (0.10-1.63)	0.203			0.46 (0.11-1.85)	0.273		
	>450	1.33 (0.62-2.85)	0.467			1.30 (0.57-2.96)	0.538		
Creatinine	≤1.3	1 (ref)				1 (ref)			
	>1.3	1.59 (1.00-2.52)	0.050			1.92 (1.20-3.06)	0.006		
Albumin	≤3.0	1 (ref)		1 (ref)		1 (ref)		1 (ref)	
	>3.0	0.21 (0.10-0.45)	<.001	0.32 (0.14-0.73)	0.0061	0.18 (0.09-0.40)	<.001	0.35 (0.15-0.79)	0.012
Nephrectomy	Open surgery	1 (ref)				1 (ref)			
	Laparoscopic	0.82 (0.56-1.20)	0.303			0.82 (0.55-1.24)	0.351		
Operative Extent	partial	1 (ref)				1 (ref)			
	radical	1.93 (1.04-3.55)	0.036			2.33 (1.11-4.89)	0.025		
pT	T1	1 (ref)		1 (ref)		1 (ref)		1 (ref)	
	T2	1.25 (0.82-1.89)	0.300	1.21 (0.77-1.9)	0.4102	1.44 (0.91-2.28)	0.116	1.42 (0.86-2.34)	0.171
	T3	2.19 (1.57-3.07)	<.001	1.83 (1.26-2.64)	0.0014	2.46 (1.69-3.58)	<.001	2.05 (1.36-3.1)	0.001

	T4+Tx	6.09 (2.99-12.39)	<.001	5.56 (2.66-11.63)	<.0001	7.82 (3.78-16.18)	<.001	6.97 (3.25-14.92)	<.001
pN	N0+Nx	1 (ref)		1 (ref)		1 (ref)		1 (ref)	
	N1	1.90 (1.13-3.18)	0.015	2.12 (1.16-3.85)	0.0141	1.81 (1.02-3.19)	0.042	2.48 (1.32-4.66)	0.005
Nuclear grade	grade 1-2	1 (ref)		1 (ref)		1 (ref)		1 (ref)	
	grade 3-4	2.01 (1.34-3.01)	0.001	1.75 (1.14-2.69)	0.0108	2.63 (1.65-4.20)	<.001	2.14 (1.3-3.51)	0.003
Sarcomatoid differentiation	yes	3.03 (1.64-5.61)	<.001			3.17 (1.66-6.07)	0.001		
Necrosis	yes	1.18 (0.79-1.76)	0.425			1.37 (0.90-2.07)	0.141		
Lymphovascular invasion	yes	1.31 (0.85-2.01)	0.220			1.42 (0.90-2.23)	0.129		
Capsular invasion	yes	1.21 (0.86-1.69)	0.273			1.19 (0.83-1.71)	0.350		

Supplementary Table 3. Survival rates for overall survival (OS) and cancer-specific survival (CSS) from 1 years to 15 years according to 3 groups

(1) Overall Survival (OS)

	MET group			LR group			BOTH group		
	survival	95% CI		survival	95% CI		survival	95% CI	
years	rate	Lower	Upper	rate	Lower	Upper	rate	Lower	Upper
1	97.70%	93.40%	100.00%	94.80%	92.38%	97.30%	98.90%	96.88%	100.00%
2	89.50%	80.20%	99.90%	86.90%	83.15%	90.80%	96.80%	93.22%	100.00%
3	79.40%	66.60%	94.60%	80.30%	75.85%	85.10%	93.20%	88.17%	98.60%
4	71.00%	56.20%	89.70%	71.10%	65.95%	76.70%	85.60%	78.41%	93.50%
5	71.00%	56.20%	89.70%	64.20%	58.65%	70.30%	77.00%	68.08%	87.00%
6	71.00%	56.20%	89.70%	59.30%	53.46%	65.80%	70.80%	61.09%	82.10%
7	71.00%	56.20%	89.70%	55.00%	49.00%	61.80%	66.00%	55.78%	78.00%
8	71.00%	56.20%	89.70%	49.00%	42.72%	56.10%	64.20%	53.88%	76.60%
9	71.00%	56.20%	89.70%	44.30%	37.87%	51.70%	64.20%	53.88%	76.60%
10	71.00%	56.20%	89.70%	43.30%	36.83%	50.90%	61.30%	50.30%	74.80%
11	71.00%	56.20%	89.70%	37.50%	30.55%	46.10%	52.50%	39.09%	70.40%
12	56.80%	34.60%	93.40%	32.40%	24.88%	42.20%	45.00%	29.49%	68.60%
13	42.60%	20.10%	90.50%	26.70%	18.35%	39.00%	45.00%	29.49%	68.60%
14	21.30%	4.40%	100.00%	20.80%	12.45%	34.80%	45.00%	29.49%	68.60%
15	21.30%	4.40%	100.00%	10.40%	2.37%	45.60%	33.70%	16.65%	68.30%

(2) Cancer-Specific Survival (CSS)

	MET group			LR group			BOTH group		
	survival	95% CI		survival	95% CI		survival	95% CI	
years	rate	Lower	Upper	rate	Lower	Upper	rate	Lower	Upper
1	97.70%	93.42%	100.00%	95.10%	92.80%	97.60%	98.90%	96.88%	100.00%
2	89.50%	80.17%	99.90%	88.90%	85.40%	92.60%	96.80%	93.22%	100.00%
3	83.20%	71.61%	96.60%	82.90%	78.60%	87.40%	93.20%	88.17%	98.60%
4	74.40%	60.02%	92.20%	75.50%	70.50%	80.80%	85.60%	78.41%	93.50%
5	74.40%	60.02%	92.20%	69.40%	63.90%	75.40%	77.00%	68.08%	87.00%

6	74.40%	60.02%	92.20%	65.60%	59.80%	71.90%	72.30%	62.76%	83.30%
7	74.40%	60.02%	92.20%	60.90%	54.70%	67.70%	67.40%	57.26%	79.30%
8	74.40%	60.02%	92.20%	55.90%	49.50%	63.30%	65.60%	55.30%	77.80%
9	74.40%	60.02%	92.20%	50.50%	43.80%	58.40%	65.60%	55.30%	77.80%
10	74.40%	60.02%	92.20%	49.50%	42.50%	57.50%	62.60%	51.60%	76.00%
11	74.40%	60.02%	92.20%	45.70%	38.30%	54.40%	53.60%	40.04%	71.70%
12	44.60%	21.15%	94.30%	39.50%	31.00%	50.30%	45.90%	30.18%	69.90%
13	44.60%	21.15%	94.30%	36.20%	26.90%	48.60%	45.90%	30.18%	69.90%
14	22.30%	4.62%	100.00%	36.20%	26.90%	48.60%	45.90%	30.18%	69.90%
15	22.30%	4.62%	100.00%	36.20%	26.90%	48.60%	34.40%	17.03%	69.70%

The highlighted colors showed the statistical differences of survival rates between MET and LR groups (blue), and BOTH and LR groups (green).
